# Apathy in rapid eye movement sleep behaviour disorder is common and under‐recognized

**DOI:** 10.1111/ene.13515

**Published:** 2017-12-14

**Authors:** T. R. Barber, K. Muhammed, D. Drew, M. Lawton, M. Crabbe, M. Rolinski, T. Quinnell, Z. Zaiwalla, Y. Ben‐Shlomo, M. Husain, M. T. M. Hu

**Affiliations:** ^1^ Nuffield Department of Clinical Neurosciences University of Oxford Oxford UK; ^2^ Oxford Parkinson's Disease Centre University of Oxford Oxford UK; ^3^ Department of Experimental Psychology University of Oxford Oxford UK; ^4^ Population Health Sciences University of Bristol Bristol UK; ^5^ Institute of Clinical Neurosciences University of Bristol Bristol UK; ^6^ Respiratory Support and Sleep Centre Papworth Hospital Cambridge UK; ^7^ Department of Clinical Neurophysiology John Radcliffe Hospital Oxford UK

**Keywords:** apathy, Parkinson's disease, prodromal, rapid eye movement sleep behaviour disorder

## Abstract

**Background and purpose:**

Apathy is an important neuropsychiatric feature of Parkinson's disease (PD), which often emerges before the onset of motor symptoms. Patients with rapid eye movement sleep behaviour disorder (RBD) have a high probability of developing PD in future. Neuropsychiatric problems are common in RBD, but apathy has not previously been detailed in this key prodromal population.

**Methods:**

Eighty‐eight patients with polysomnographically proven RBD, 65 patients with PD and 33 controls were assessed for apathy using the Lille Apathy Rating Scale. Cognition and depression were also quantified. The sensitivity of the Unified Parkinson's Disease Rating Scale screening questions for apathy and depression was calculated.

**Results:**

A total of 46% of patients with RBD were apathetic, compared with 31% of patients with PD in our sample. Most patients with RBD with depression were apathetic but more than half of apathetic patients were not depressed. The sensitivity of the single Unified Parkinson's Disease Rating Scale screening question was only 33% for mild apathy and 50% for severe apathy.

**Conclusions:**

Apathy is common in RBD and is underestimated by a single self‐report question. Recognition of apathy as a distinct neuropsychiatric feature in RBD could aid targeted treatment interventions and might contribute to the understanding of prodromal PD.

## Introduction

Apathy is a common and debilitating feature of Parkinson's disease (PD) and dementia with Lewy bodies (DLB), which impacts on the quality of life of both patients and carers 1, 2, 3. Characterized by pathological lack of motivation, it manifests as a reduction in goal‐directed thoughts and behaviours, with or without emotional blunting 4. Although it frequently coexists with other neuropsychiatric conditions, such as depression and cognitive impairment, apathy is increasingly recognized as a distinct syndrome with different aspects that are not always present 5, 6. It is not equivalent to anhedonia, as patients with apathy are still able to enjoy activities if they are prompted to act.

As with many non‐motor features of PD and DLB, apathy is often reported retrospectively to occur before the onset of motor symptoms 7. Despite this, the syndrome has never been formally assessed in the prodromal period. As well as furthering understanding of prodromal PD/DLB, recognizing apathy if it occurs early might lead to interventions with specific psychological and pharmacological therapies to improve quality of life in this phase of disease.

Patients with rapid eye movement sleep behaviour disorder (RBD) have a long‐term risk exceeding 80% of developing an alphasynuclein‐related neurodegenerative disorder, with the majority converting to either PD or DLB 8. Patients with RBD therefore present a valuable opportunity to study the prodromal phase of these disorders. Although apathy is recognized in RBD, most studies rely on single‐question self‐report measures 9, 10, which may fail to capture the full spectrum of the condition. It is now recognized that there might be many components of apathy, including deficits in self‐awareness and emotional responses as well as action initiation and intellectual curiosity 11.

One recent study of RBD demonstrated that depression, anxiety and cognitive impairment are particularly common, suggesting that RBD might represent the prodromal phase of a subtype of alphasynucleinopathy with a more severe neuropsychiatric phenotype 9. Such parkinsonian non‐motor features may have a role in risk‐stratification models to predict conversion to PD/DLB 12; accurately establishing the presence and severity of apathy in RBD is therefore an important objective.

Apathy might be of particular relevance in prodromal PD/DLB because of the role of dopamine in neural pathways underlying reward and effort sensitivity that appear to be key to motivation 13. Dopaminergic neurodegeneration proceeds for many years prior to the onset of motor parkinsonism, with more than 50% of substantia nigra neurons having been lost by the time that motor symptoms emerge 14. The mesolimbic pathway is also vulnerable to degeneration in PD 15, and may have a more specific role in the development of neuropsychiatric manifestations 16. Although neurodegeneration in the mesolimbic system has not been clearly established in prodromal disease, it is plausible that deficits caused by early involvement of this pathway in the disease process may be detectable in patients with RBD.

The aim of this study was to investigate the frequency and severity of apathy in a large cohort of patients with RBD and to compare the results with patients with PD and healthy controls. We used the Lille Apathy Rating Scale (LARS) to assess apathy as it provides the most detailed and structured evaluation of any rating system, including subdomains of apathy as well as an overall measure of apathy severity 11. Unlike some other apathy ratings, it does not rely on subjective reports of caregivers and therefore has high inter‐rater consistency and excellent accuracy compared with clinical judgement 17. It can effectively distinguish apathy from depression and has been validated in established PD with and without dementia 11. In addition, we sought to explore the overlap between apathy, cognition and depression, and also to assess the value of a commonly used apathy screening question from the Unified Parkinson's Disease Rating Scale (UPDRS) I.

## Methods

### Participants

Patients with RBD were recruited from an established UK research cohort described elsewhere 18. They were recruited to the study from sleep centres at the John Radcliffe Hospital, Oxford and Papworth Hospital, Cambridge, UK. Patients with PD were recruited separately from neurology clinics in the Oxfordshire area. Control participants were recruited from a volunteer database and screened to exclude those with neurological conditions. All RBD diagnoses were made by polysomnography according to International Classification of Sleep Disorders criteria 19. Patients with RBD were examined by a neurologist to exclude the presence of PD and other neurological disorders. Patients with dementia, identified using a combination of cognitive testing {Montreal Cognitive Assessment (MOCA) 20}, informant ratings (using the Informant Questionnaire on Cognitive Decline in the Elderly 21) and clinical judgement, were not included. We also excluded patients with RBD with concomitant moderate or severe obstructive sleep apnoea (apnoea hypopnea index ≥ 15). The study was approved by the Oxfordshire Research Ethics Committee and all participants provided informed, written consent.

### Assessments

Apathy was assessed using the LARS 11. The scale includes 33 items, divided into nine domains. The global score ranges from −36 to +36, with a higher score representing a greater degree of apathy. We used the following cut‐off values for categorical classification as indicated in the validation of the scale: no apathy, ≤−22; mild apathy, −21 to −17; moderate to severe apathy, ≥−16. Depression was assessed using the Beck Depression Inventory (BDI) 22, with a score >13 indicative of depression. Cognitive impairment was assessed using the MOCA 20. A threshold for cognitive impairment of <24 on the MOCA was used, as this value optimizes the specificity and positive predictive value for mild cognitive impairment 23. The Epworth Sleepiness Scale^24^ was used to assess daytime sleepiness in subjects with RBD, with the standard threshold of >10 indicating excessive daytime sleepiness. Participants with PD and RBD also completed the Movement Disorders Society UPDRS 25, including the motor assessment (part III) and the screening questions for apathy (part I, question 1.5) and depression (part I, question 1.3). Data from polysomnography were taken from the diagnostic studies and were therefore not collected at the same time as clinical assessments.

### Statistical analysis

Between‐groups comparisons for continuous variables were made using linear regression and for dichotomous variables using logistic regression, with gender (as a categorical variable), age, BDI and MOCA scores (as continuous variables) included as covariates where indicated to control for any between‐group differences that may also impact on apathy ratings. Comparisons of LARS scores within patient groups where other variables were not adjusted for were made using the Mann–Whitney *U*‐test or Kruskal–Wallis test as appropriate, and the chi‐square test was used for categorical outcomes. Pearson correlation coefficients were used to assess relationships between two continuous variables. Sensitivity, specificity and positive predictive value of the UPDRS part I screening questions were calculated using standard formulae, with a UPDRS I response >0 considered as a positive screening result. Only cases of RBS and PD, but not controls, had a UPDRS assessment. Subgroups for the sensitivity analyses were generated using a frequency‐matching process.

## Results

Assessments were completed in 88 patients with RBD, 65 age‐matched patients with PD and 33 healthy controls. Patients with RBD had a mean duration of 8.5 (SD 6.7) years since symptom onset and 3.0 (SD 2.5) years since polysomnographic diagnosis. Patients with PD had mean disease duration since diagnosis of 4.4 (SD 4.0) years and mean total UPDRS score of 41.8 (SD 17.5). A total of 10 patients with PD were drug naive, 47 were receiving treatment with L‐DOPA, 26 with a dopaminergic agonist and 30 were taking other PD medications (monoamine oxidase inhibitors, catechol‐O‐methyl transferase inhibitors or amantadine).

The results of clinical assessments are shown in Table 1. Comparisons between groups for all assessment scores were adjusted for age and gender. In keeping with previous reports, measures of cognition and depression were significantly worse in patients with RBD compared with controls. Apathy classed as mild or worse was present in 45.5% [95% confidence interval (CI), 34.8–56.4%] of subjects with RBD, compared with 30.8% (95% CI, 19.9–43.4%) of patients with PD and only 3.2% (95% CI, 0.1–16.7%) of control participants. Moderate to severe apathy was present in 15.9% (95% CI, 9.0–25.2%) of patients with RBD, 18.5% (95% CI, 9.9–30.0%) of patients with PD and 3.2% (95% CI, 0.1–16.7%) of control subjects. Mean total LARS scores in patients with RBD were significantly worse than in controls, and similar to those in patients with PD. Breakdown of the LARS scores revealed that patients with RBD were impaired across all subdomains compared with controls except the one that seeks to measure emotional apathy where their worse score was consistent with chance variability. Compared with patients with PD, action initiation and self‐awareness LARS scores were actually worse in cases of RBD.

**Table 1:** Demographic and neuropsychiatric characteristics

	Control (*n* = 33)	RBD (*n* = 88)	PD (*n* = 65)	*P* value RBD vs. control	*P* value RBD vs. PD
Age (years)	68.4 (8.94)	66.9 (7.62)	66.4 (5.65)	0.31	0.70
Male (%)	45.5	94.3	72.3	**<0.001**	**0.001**
UPDRS score	n/a	5.25 (4.02)	41.8 (17.5)	n/a	**<0.001**
MOCA score	28.3 (1.44)	24.9 (3.08)	27.6 (2.30)	**<0.001**	**<0.001**
BDI score	4.91 (4.91)	9.40 (7.68)	10.2 (7.01)	**0.01**	0.48
Total LARS score	−29.1 (4.33)	−21.0 (6.00)	−23.3 (6.85)	**<0.001**	0.10
LARS intellectual curiosity score	−3.30 (0.63)	−2.37 (0.96)	−2.29 (1.07)	**<0.001**	0.41
LARS emotion score	−2.62 (1.03)	−1.98 (1.13)	−2.32 (1.25)	0.10	0.19
LARS action initiation score	−3.76 (0.47)	−2.77 (1.00)	−3.25 (0.89)	**<0.001**	**0.005**
LARS self‐awareness score	−3.12 (1.32)	−2.03 (1.52)	−2.97 (1.32)	**0.002**	**<0.001**
Mild apathy (%; LARS score ≥−21)	3.0	45.5	30.8	**0.008**	0.23
Moderate to severe apathy (%; LARS score ≥−16)	3.0	15.9	18.5	0.22	0.40

BDI, Beck Depression Inventory; LARS, Lille Apathy Rating Scale; MOCA, Montreal Cognitive Assessment; n/a, not applicable; PD, Parkinson's disease; RBD, rapid eye movement sleep behaviour disorder; UPDRS, Unified Parkinson's Disease Rating Scale. Values are mean (SD) for continuous variables (age, MOCA, BDI, LARS total and subdomain scores) and percentages for dichotomous variables (gender, mild and severe apathy categories); *P* values in bold indicate statistically significant group differences at the <0.05 level.

Using a LARS score ≥−21 as indicative of apathy, the sensitivity of the UPDRS I apathy screening question in patients with RBD was only 33% (95% CI, 19–49%), specificity was 85% (95% CI, 72–94%), positive predictive value was 65% (95% CI, 41–85%), negative predictive value was 60% (95% CI, 48–72%), positive likelihood ratio was 2.2 and negative likelihood ratio was 0.79. The sensitivity for moderate to severe apathy (LARS score ≥−16) was slightly improved at 50% (95% CI, 23–77%) but with wide CIs due to relatively small numbers. However, the sensitivity of the UPDRS screening question for depression in RBD (defined as BDI score > 13) was 80% (95% CI, 63–98%).

The extent of apathy in patients with RBD was not explained by excessive daytime sleepiness. There was no significant correlation between LARS scores and Epworth Sleepiness Scale scores in patients with RBD as a whole (*r* = 0.10, *P* = 0.34) or in apathetic patients with RBD (*r* = 0.14, *P* = 0.40). Furthermore, there was no evidence for apathy occurring as a result of disturbed sleep itself, as LARS scores did not correlate with polysomnographic measures of sleep quality (total sleep time: *r* = −0.04, *P* = 0.76; sleep efficiency: *r* = 0.16, *P* = 0.19; percentage slow‐wave sleep: *r* = 0.13, *P* = 0.27).

Forty patients with RBD (45%) were taking clonazepam at the time of testing as treatment for RBD symptoms. Sedation commonly occurs as a side‐effect of clonazepam and may conceivably contribute to symptoms of apathy. Although patients with RBD taking clonazepam had higher total LARS scores (mean −19.8) than those not taking clonazepam (mean −22.0), this was consistent with chance (*P* = 0.07). Furthermore, patients with RBD not taking clonazepam were still significantly more apathetic than healthy controls (*P* < 0.001), and showed similar levels of apathy to patients with PD (*P* = 0.12), meaning that apathy in RBD cannot be fully explained as a side‐effect of clonazepam.

**Figure 1:**
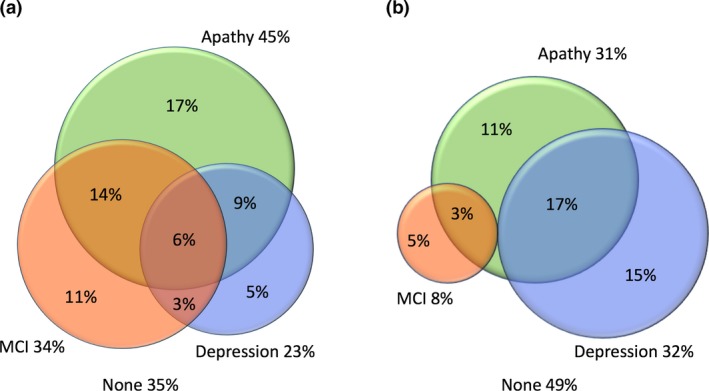
Overlap between apathy, depression and mild cognitive impairment (MCI) in patients with (a) rapid eye movement sleep behaviour disorder (*n* = 88) and (b) Parkinson's disease (*n* = 65). Apathy is defined as Lille Apathy Rating Scale score ≥−21, cognitive impairment as Montreal Cognitive Assessment score <24 and depression as Beck Depression Inventory score >13.

The overlap between apathy, depression and cognitive impairment in patients with RBD and PD is shown in Fig. 1. Although these features frequently coexisted, there was clear dissociation between them. A total of 33% of patients with RBD had one condition alone and, crucially, two‐thirds of those with apathy were not depressed. Patients with PD in this sample actually had less cognitive impairment, but the dissociation between apathy and depression was similar to that seen in RBD.

To explore the relationship between depression and apathy further, we examined the correlation between LARS and BDI scores in the three patient groups. In control participants there was no correlation (*r* = 0.0, *P* = 0.99), whereas moderate correlations were present in patients with RBD (*r* = 0.29, *P* = 0.007) and PD (*r* = 0.46, *P* < 0.001). Amongst apathetic patients with RBD, however, there was no significant correlation between the severity of depression and apathy (*r* = 0.10, *P* = 0.53). Moreover, patients with RBD with depression were not significantly more apathetic than non‐depressed patients with RBD (mean LARS score −19.1 vs. −21.6, *P* = 0.12). When individuals with depression (BDI score > 13) were excluded in a sensitivity analysis, patients with RBD remained significantly more apathetic than controls (Table S1).

In the PD group, patients with depression were significantly more apathetic than those without depression (mean LARS score −19.9 vs. −25.0, *P* = 0.01). When excluding all patients with depression, patients with RBD were significantly more apathetic than patients with PD (Table S1). These data suggest that, although there is an association between apathy and depression, particularly in PD, apathy in patients with RBD cannot be explained simply by the presence of depression.

There were weak correlations between LARS and MOCA scores in all of the groups (control: *r* = 0.20, *P* = 0.27; RBD: *r* = −0.17, *P* = 0.11; PD: *r* = −0.17, *P* = 0.17), which were consistent with chance. There was a trend towards more severe apathy in patients with RBD with cognitive impairment (MOCA score < 24) than in those without, but this was consistent with random variability (mean LARS score −19.5 vs. −21.8, *P* = 0.08).

Despite this evidence of dissociation between apathy, depression and cognitive impairment, there remains some degree of overlap in their clinical features. In order to control for this, we repeated the comparisons between RBD versus PD and RBD versus controls using a linear regression model that included BDI and MOCA scores as covariates in addition to age and gender. The results of this are shown in Table 2. The same‐group differences in LARS total scores and subdomains were observed using this analysis. To further control for group differences, we undertook additional sensitivity analyses using two subgroups of patients, the first with subjects matched for age and MOCA scores, and the second with subjects matched for gender. These are shown in the Supporting Information (Tables S2 and S3). The same‐group differences in apathy scores remained, with patients with RBD always significantly more apathetic than controls and of similar apathy severity to patients with PD.

**Table 2:** Apathy scores with group comparisons adjusted for age, gender, Beck Depression Inventory (BDI) and Montreal Cognitive Assessment (MOCA) scores

	Control (*n* = 33)	RBD (*n* = 88)	PD (*n* = 65)	*P* value RBD vs. control	*P* value RBD vs. PD
Total LARS score	−29.1 (4.33)	−21.0 (6.00)	−23.3 (6.85)	**0.002**	0.35
LARS intellectual curiosity score	−3.30 (0.63)	−2.37 (0.96)	−2.29 (1.07)	**0.01**	0.45
LARS emotion score	−2.62 (1.03)	−1.98 (1.13)	−2.32 (1.25)	0.44	0.57
LARS action initiation score	−3.76 (0.47)	−2.77 (1.00)	−3.25 (0.89)	**0.02**	**0.05**
LARS self‐awareness score	−3.12 (1.32)	−2.03 (1.52)	−2.97 (1.32)	**0.02**	**0.02**
Mild apathy (%; LARS score ≥−21)	3.0	45.5	30.8	**0.05**	0.41
Moderate to severe apathy (%; LARS score ≥−16)	3.0	15.9	18.5	0.76	0.17

LARS, Lille Apathy Rating Scale; PD, Parkinson's disease; RBD, rapid eye movement sleep behaviour disorder. Values are mean (SD) for continuous variables (age, MOCA, BDI, LARS total and subdomain scores) and percentages for dichotomous variables (gender, mild and severe apathy categories); *P* values in bold indicate statistically significant group differences at the <0.05 level.

One potential confounding variable in the PD group is the use of dopaminergic medication. Dopamine receptor agonists (DAs), in particular, have been shown to reduce apathy and may therefore contribute to lower apathy ratings in the participants with PD 26. Table 3 shows the same variables as Table 1, with patients with PD divided into three groups according to treatment: drug naive, those taking DAs (with or without L‐DOPA) and those taking L‐DOPA without agonists. Patients with PD taking DAs were less apathetic than drug‐naive patients and those taking L‐DOPA, although there was no significant effect of treatment type (Kruskal–Wallis test across patients with PD by treatment type, *P* = 0.21). However, patients with PD taking DAs were significantly less apathetic than patients with RBD (mean LARS score −24.4 vs. −21.0, *P* = 0.003).

**Table 3:** Distribution of apathy scores in patients with Parkinson's disease (PD) according to treatment type

	Control (*n* = 33)	RBD (*n* = 88)	PD drug naive (*n* = 10)	PD agonist ±L‐DOPA (*n* = 26)	PD L‐DOPA without DA (*n* = 26)	*P* value across three PD groups	*P* value patients with RBD vs. PD not taking DA
Age (years)	68.4 (8.94)	66.9 (7.62)	64.8 (7.19)	65.8 (5.70)	67.3 (5.10)	0.37	0.98
Male (%)	45.5	94.3	50	69	81	0.18	**0.003**
MOCA score	28.3 (1.44)	24.9 (3.08)	25.9 (3.14)	28.3 (1.93)	27.7 (2.09)	**0.04**	**<0.001**
BDI score	4.91 (4.91)	9.40 (7.68)	11.1 (7.79)	9.0 (7.07)	11.0 (6.59)	0.44	0.29
Total LARS score	−29.1 (4.33)	−21.0 (6.00)	−23.2 (7.61)	−24.4 (6.84)	−21.8 (6.50)	0.21	0.39
LARS intellectual curiosity score	−3.30 (0.63)	−2.37 (0.96)	−2.53 (1.24)	−2.37 (0.94)	−2.12 (1.12)	0.42	0.63
LARS emotion score	−2.62 (1.03)	−1.98 (1.13)	−1.85 (1.55)	−2.56 (1.40)	−2.17 (0.94)	0.10	0.79
LARS action initiation score	−3.76 (0.47)	−2.77 (1.00)	−3.25 (0.95)	−3.48 (0.66)	−3.00 (1.07)	0.28	0.23
LARS self‐awareness score	−3.12 (1.32)	−2.03 (1.52)	−2.90 (1.29)	−2.88 (1.48)	−2.96 (1.25)	0.97	**0.006**
Mild apathy (%; LARS score ≥−21)	3.0	45.5	30	19	42	0.20	0.74
Severe apathy (%; LARS score ≥−16)	3.0	15.9	20	12	27	0.37	0.16

BDI, Beck Depression Inventory; DA, dopamine receptor agonist; LARS, Lille Apathy Rating Scale; MOCA, Montreal Cognitive Assessment; RBD, rapid eye movement sleep behaviour disorder; UPDRS, Unified Parkinson's Disease Rating Scale. Values are mean (SD) for continuous variables (age, MOCA, BDI, LARS total and subdomain scores) and percentages for dichotomous variables (gender, mild and severe apathy categories); *P* values in bold indicate statistically significant group differences at the <0.05 level.

To remove any effect of DAs on overall group apathy ratings, we performed a comparison between patients with RBD and PD excluding those taking DAs (Table 3). There remained no significant difference in overall LARS scores. With respect to the whole‐group comparison, the difference in action initiation subscores was no longer significant, but patients with RBD remained significantly worse in the self‐awareness subscore. We conclude that some of the apparent differences between RBD and PD may reflect dopaminergic therapy (making the PD group appear less apathetic) but, even without the effect of DAs, patients with RBD are at least as apathetic as patients with PD.

Within the PD group, there was evidence of an association between apathy and disease severity; UPDRS motor scores (part III) were positively correlated with LARS scores (*r* = 0.29, *P* = 0.02) and this effect remained after adjusting for age (*P* = 0.02). There was no such relationship in patients with RBD (*r* = 0.03, *P* = 0.75) but, as these patients do not have significant motor parkinsonism, UPDRS III score is not a sensitive measure of prodromal disease stage. To determine whether apathy in RBD is associated with increased risk of developing PD will require long‐term follow‐up.

## Discussion

In the first study to quantify the extent and severity of apathy in RBD, we have shown that the condition is both common and under‐recognized. Approximately half of all patients with RBD in this cohort were affected by apathy, a prevalence similar to that seen in established PD in our sample. The presence of apathy in RBD was not explained by excessive daytime sleepiness, poor sleep quality or sedative medication. Amongst subdomains of apathy, all were impaired in RBD except emotional responses, suggesting that problems with motivation for action, intellectual curiosity and self‐awareness, rather than affect, predominate in patients with RBD.

Our data suggest that the single screening question for apathy in the UPDRS assessment has modest sensitivity, capturing only one‐third of all apathetic patients with RBD and just half of those with moderate to severe apathy, and a relatively weak positive likelihood ratio. This is in contrast to the UPDRS screening question for depression, which was 80% sensitive in this study. There are a number of possible explanations for this. First, the UPDRS question asks only about ‘indifference to doing activities or being with people’ and therefore may not capture other subdomains of apathy. Secondly, apathy might be associated with reduced insight compared with depression as a result of impaired self‐awareness, which would limit the sensitivity of self‐report measures. Finally, it may be accounted for in part by the high rate of cognitive impairment in RBD, as studies of apathy in other populations have shown discordance between self‐report and clinician‐rated measures of apathy in cognitively impaired individuals 27.

Although there is significant overlap between apathy, depression and cognitive impairment, these are clearly dissociable; only 33% of apathetic patients with RBD were depressed and less than half had cognitive impairment. Furthermore, differences in the degree of apathy between patient groups could not be explained by differences in cognition and depression. This evidence of apathy as a distinct neuropsychiatric syndrome highlights the importance of actively looking for apathy even when other neuropsychiatric features are absent. It also raises the possibility that, where apathy coexists with depression and/or cognitive impairment, these are distinct manifestations of a common underlying neurodegenerative process rather than a single neuropsychiatric condition.

The association between apathy and severity of motor disease in patients with PD suggests that apathy may be related to disease progression. The fact that patients with RBD were as apathetic as those with PD, in our sample, is not inconsistent with this, despite them being at an earlier neurodegenerative stage. Accumulating evidence suggests that patients with PD who develop RBD first might represent a distinct subtype of disease with a more severe non‐motor phenotype, and that many of these non‐motor features emerge prior to the onset of motor parkinsonism 28, 29. In keeping with this hypothesis, we recently demonstrated that a wide range of non‐motor features are as severe in RBD as they are in newly diagnosed PD, and that depression and anxiety are worse in RBD 9. In a separate study of patients with established PD, we found that apathy was significantly more common in patients with PD with concomitant RBD than in those without 30. The co‐occurrence of RBD and other non‐motor features might be a consequence of more widespread Lewy body pathology in these patients, affecting brain regions involved in the regulation of sleep, cognition and mood in addition to motor control 31.

A potential underlying mechanism for apathy in RBD is the degeneration of dopaminergic neurons involved in motivation and reward/effort‐based decision‐making pathways. In established PD there is considerable evidence for the importance of dopamine dysfunction in apathy. Functional imaging studies have demonstrated greater dopaminergic deficits in apathetic patients with PD than in patients with PD without apathy 32, 33, and dopaminergic agonists have been proven to partially alleviate symptoms of apathy in PD 26. The ventral striatum and dorsal anterior cingulate cortex appear to be key regions involved in generating motivated behaviour 34, and dysfunction in striatofrontal functional connectivity has been shown to associate with apathy in patients with PD 35. Although the dopaminergic motor system displays considerable capacity for compensation in the prodromal phase of PD, it is possible that deficits in these motivational pathways emerge at an earlier stage of dopamine depletion. Future work with functional neuroimaging in patients with RBD will be important to shed light on the relationship between apathy and dysfunction in these brain regions.

Recognition of apathy as a distinct neuropsychiatric condition is important as it may guide the choice of both psychological and pharmacological interventions. Although few interventional trials have been conducted on apathy, there is evidence that, in depressed patients with motivational problems, behavioural activation techniques may be superior to standard cognitive behavioural therapy, and medications with dopaminergic effects may have greater efficacy than selective serotonin reuptake inhibitors 36. This might be particularly relevant in patients with RBD when a dopaminergic deficit is revealed, e.g. using nuclear imaging. Further studies are needed to identify the most effective treatments for apathy.

An important finding to be established from future follow‐up of our cohort will be the extent to which apathy predicts conversion to neurodegenerative disorders. Depression (with or without anxiety) and excessive daytime somnolence have been shown to increase risk of conversion in patients with RBD, and these features are included in the Movement Disorders Society Research Criteria for prodromal PD 12. Longitudinal study of conversion from RBD to PD in our participants will be important in establishing whether apathy may form an equally important part of such risk‐stratification models.

A limitation of our study should be noted. Accurately matching across all three groups for age, gender, depression and cognition was not feasible given the way that these variables are differentially distributed amongst the populations studied. RBD is diagnosed predominantly in males 28, and it is expected that measures of cognition and depression will be worse in RBD than controls 37, 38. The fact that our RBD group had lower MOCA scores than the PD group is perhaps more surprising, but it should be noted that patients with RBD are as likely to develop DLB as PD, and the extent of cognitive impairment in patients with RBD may reflect this 39. We have controlled as much as possible for the group differences by including all variables in our regression model where appropriate, and by performing sensitivity analyses, but it is still possible that some of the variation in apathy is accounted for by differences in these other characteristics.

In summary, we have shown that apathy is a common and under‐recognized feature of RBD and should be actively sought as part of the clinical phenotype of this disorder. Longitudinal follow‐up will establish the extent to which apathy may herald conversion to PD or a related neurodegenerative disorder.

## Disclosure of conflicts of interest

The authors declare no financial or other conflicts of interest.

## Supporting information


**Table S1.** Subjects with depression excluded (group comparisons adjusted for age and gender)
**Table S2.** Groups matched for Montreal Cognitive Assessment scores (group comparisons adjusted for age and gender)
**Table S3.** Groups matched for gender (group comparisons adjusted for age)
