# Supplementary material for: Apathy in rapid eye movement sleep behaviour disorder is common and under‐recognized
Source: Eur J Neurol. 2017 Dec 14;25(3):469–e32. doi: 10.1111/ene.13515 (PMC5838543; doi:10.1111/ene.13515)
Supplement: Supplementary file 1 — Table S1. Subjects with depression excluded (group comparisons adjusted for age and gender) Table S2. Groups matched for Montreal Cognitive Assessment scores (group comparisons adjusted for age and gender) Table S3. Groups matched for gender (group comparisons adjusted for age) [file ENE-25-469-s001.docx]

Supplementary table 1. Subjects with depression excluded (group comparisons adjusted for age and gender)

|  | Control  N=31 | RBD  N=66 | PD  N=43 | p-value  RBD vs Control | p-value  RBD vs PD |
| --- | --- | --- | --- | --- | --- |
| Age | 69.0 (8.88) | 67.2 (7.63) | 66.6 (4.46) | 0.27 | 0.66 |
| % male | 45.2 | 92.4 | 72.1 | <0.001 | 0.007 |
| MOCA | 28.3 (1.47) | 26.5 (2.98) | 28.0 (2.25) | **<0.001** | **<0.001** |
| LARS | -29.0 (4.44) | -21.64 (5.75) | -25.0 (5.63) | **<0.001** | **0.01** |
| Mild apathy (%) | 3.2 | 39.4 | 20.9 | **0.03** | 0.15 |
| Moderate to severe apathy (%) | 3.2 | 9.1 | 11.6 | 0.77 | 0.39 |

Supplementary table 2. Groups matched for MOCA scores (group comparisons adjusted for age & gender)

|  | Control  N=28 | RBD  N=34 | PD  N=54 | p-value  RBD vs Control | p-value  RBD vs PD |
| --- | --- | --- | --- | --- | --- |
| Age | 66.5 (7.93) | 65.9 (6.51) | 66.0 (5.53) | 0.70 | 0.95 |
| % male | 42.9 | 94.1 | 77.8 | **<0.001** | 0.06 |
| MOCA | 28.3 (1.48) | 27.9 (1.09) | 28.2 (1.40) | 0.45 | 0.48 |
| BDI | 4.71 (5.28) | 10.0 (7.73) | 10.5 (7.13) | **0.01** | 0.75 |
| LARS | -29.1 (4.34) | -22.0 (5.05) | -23.2 (7.22) | **<0.001** | 0.47 |
| Mild apathy (%) | 3.6 | 41.2 | 31.5 | **0.02** | 0.53 |
| Moderate to severe apathy (%) | 3.6 | 11.8 | 20.4 | 0.40 | 0.24 |

|  | Control  N=16 | RBD  N=88 | PD  N=50 | P-value  RBD vs Control | P-value  RBD vs PD |
| --- | --- | --- | --- | --- | --- |
| Age | 70.6 (9.00) | 66.9 (7.62) | 66.1 (5.78) | 0.06 | 0.56 |
| % male | 94 | 94 | 94 | 0.94 | 0.93 |
| MOCA | 28.3 (1.25) | 24.9 (3.08) | 27.6 (1.95) | **<0.001** | **<0.001** |
| BDI | 4.69 (4.72) | 9.40 (7.68) | 10.32 (7.06) | **0.03** | 0.52 |
| Total LARS | -29.0 (4.94) | -21.0 (6.00) | -22.6 (6.93) | **<0.001** | 0.15 |
| Mild apathy % | 6.3 | 45.5 | 36.0 | **0.02** | 0.26 |
| Moderate to severe apathy (%) | 6.3 | 15.9 | 22.0 | 0.34 | 0.38 |

Supplementary table 3. Groups matched for gender (group comparisons adjusted for age)
